# Psoriatic Juvenile Idiopathic Arthritis Associated with Uveitis: A Case Report

**DOI:** 10.1155/2013/595890

**Published:** 2013-09-29

**Authors:** Davide Moretti, Ilaria Cianchi, Gaia Vannucci, Rolando Cimaz, Gabriele Simonini

**Affiliations:** Rheumatology Unit, Department of Paediatrics, University of Florence, Anna Meyer Children's Hospital, Viale Pieraccini 24, 50139 Firenze, Italy

## Abstract

According to the definition proposed by the International League of Associations for Rheumatology (ILAR), juvenile idiopathic arthritis (JIA) is defined as an arthritis of unknown etiology, starting under 16 years of age and lasting for at least 6 weeks, once other known conditions have been excluded. JIA represents the most common chronic rheumatic disease of childhood and is considered an important cause of short- and long-term acquired disability in children. It is currently estimated that psoriatic JIA represents up to 10% of all JIA subtypes, and chronic uveitis may occur in 10 to 15% of children with psoriatic JIA. In this report we describe a case of psoriatic JIA complicated by uveitis, in a child failing previous treatments with nonsteroidal anti-inflammatory drugs, methotrexate, and etanercept. Finally, adalimumab was prescribed, which led to sustained clinical remission in both arthritis and uveitis.

## 1. Background

According to the definition proposed by the International League of Associations for Rheumatology (ILAR), juvenile idiopathic arthritis (JIA) is defined as an arthritis of unknown etiology, starting before 16 years of age, and lasting for at least 6 weeks, once other known conditions have been excluded [1]. JIA represents the most common chronic rheumatic disease of childhood and is considered an important cause of short- and long-term acquired disability in children [2]. JIA encompasses seven different subcategories based on the predominant clinical manifestations and laboratory features seen in the first 6 months of disease. Its prevalence is unknown and varies considerably among populations, depending on race, immunogenetic susceptibility, and environmental influences. Currently, the annual incidence of JIA is estimated at around 100 new cases per 1,000,000 population [3].

JIA is considered an autoimmune disease, potentially resulting from an abnormal immunologic response caused or triggered by environmental factors such as infection or trauma in a genetically predisposed subject. Adaptive immune activation against self-epitopes has been suggested, and a typical synovial membrane inflammation has been observed [4–6]. The synovia shows pronounced hyperplasia of the lining layer, along with an exuberant infiltration of the sublining layer with mononuclear cells, including memory T cells, B cells, macrophages, dendritic cells, and plasma cells [2].

Psoriatic JIA represents up to 10% of all JIA subtypes and has a predilection for females. Anti-nuclear antibodies (ANAs) are positive in more than 50% of affected patients. According to the ILAR classification, psoriatic JIA has to satisfy the criteria shown in the following [1].

*ILAR Inclusion and Exclusion Criteria for Psoriatic JIA [1]:*

*ILAR Inclusion Criteria*. Arthritis is associated with psoriasis or two of the following:

(1) dactylitis,
(2) nail pitting,
(3) onycholysis,
(4) psoriasis in a first degree relative.

*ILAR Exclusion Criteria*. Consider the following:

(1) arthritis in HLA-B27-positive males beginning after the age of 6 years,
(2) ankylosing spondylitis, enthesitis-related arthritis, sacroiliitis with inflammatory bowel disease, Reiter's syndrome, acute anterior uveitis, or history of one of these in a first-degree relative,
(3) presence of IgM rheumatoid factor on at least two occasions more than 3 months apart,
(4) presence of systemic arthritis.

Arthritis precedes the psoriatic cutaneous manifestations in more than 60% of cases, sometimes years earlier [7], and usually presents in an asymmetric oligoarthritis pattern. Monoarthritis is relatively common at the onset, with isolated involvement of knee and small joints of hands and feet. Without effective therapy, progression to the polyarticular form occurs in 60–80% of children. Dactylitis, swelling of the whole digit (i.e., extending beyond the borders of the joint), with the subsequent typical “sausage digit” appearance, is observed in 20–40% of affected patients. Cutaneous manifestations are often represented by nail pitting (diffused or confluent multiple pits localized on the nail surface), discoloration, and onycholysis, involving all or part of the nail.

Younger ANA positive patients are at highest risk of developing ocular complications such as chronic uveitis, which may occur in 10 to 15% of children with psoriatic JIA [8].

## 2. Case Presentation

At the age of 2 years and 6 months, a Caucasian male with no significant past medical or family history presented at our emergency department with progressive painful swelling of the left knee, along with morning stiffness and limited range of motion. Physical examination revealed arthritis of the left knee, and laboratory findings showed a mild increase in C-reactive protein (CRP) level and erythrocyte sedimentation rate (ESR). An ultrasound confirmed intra-articular effusion. Symptomatic therapy with a nonsteroidal anti-inflammatory drug (NSAID) was prescribed, and the child was referred to our rheumatology unit for further investigation and followup. Our first evaluation confirmed both previous clinical and laboratory findings. The ANA titre was positive (1 : 320), HLA-B27 was negative, and slit lamp examination showed no signs of uveitis. One month later, while still receiving NSAIDs, three additional joints were active (right knee, right ankle, and fourth right proximal interphalangeal joint), while ophthalmologic examination with slit lamp revealed anterior chamber inflammation of the right eye. Due to the progressive and additive course of joint disease together with the eye involvement, methotrexate (MTX; 15 mg/m^2^) was administered in association with the NSAIDs therapy. Sustained clinical benefit was rapidly obtained, with a complete resolution of articular manifestations and uveitis, associated with normalization of inflammatory parameters. After 2 years of clinical remission on this therapy, psoriasiform lesions appeared on elbows and knees, and a diagnosis of psoriasis was made. A few months later, while still receiving MTX, a new episode of arthritis involving both knees and the right ankle occurred. Laboratory findings again showed increased ESR and CRP levels, and slit lamp examination revealed a new flare of uveitis. Considering the poor clinical control achieved with the current medications, we decided to add a biologic modifier to MTX therapy, and so etanercept, a tumour necrosis factor (TNF) inhibitor, was administered subcutaneously at the dose of 0.4 mg/Kg twice weekly. Clinical remission was rapidly obtained, with absence of joint swelling and uveitis flares. In consideration of the persistent clinical benefit reached through the combined therapy, we decided to stop the MTX administration after 1 year from its introduction due to persistent elevation of liver functions tests. Six months later, slit lamp exam revealed again new signs of anterior chamber uveitis activity, this time affecting both eyes. After one month, a severe arthritis flare occurred, with an aggressive polyarticular course. In consideration of the lack of control obtained through the Enbrel administration and according to the current literature, which showed the efficacy and safety of adalimumab in the treatment of uveitis in JIA patients who failed previous therapies, we then decided to switch from etanercept to subcutaneous adalimumab (Humira, AbbVie Inc. Chicago, IL), which was administered in monotherapy at 24 mg/m^2^ every other week. After 3 months' followup, the child experienced complete remission of arthritis and uveitis, which has lasted for two years (up to the latest visit). No adverse reactions occurred during treatment.

## 3. Discussion

We present a case of psoriatic JIA complicated by uveitis, failing previous treatments with NSAID, MTX, and etanercept.

American College of Rheumatology (ACR) JIA treatment recommendations are based on a step-up approach, requiring the subsequent use of drugs with greater power once the previous treatment has failed [9]. Regarding systemic therapy, NSAIDs are prescribed as first-line treatment of symptoms, followed by disease-modifying antirheumatic drugs (DMARDs), such as MTX, and low-dose oral corticosteroids, if needed, for short periods. In case of an incomplete response after 3–6 months, biologic agents, beginning with an anti-TNF*α* agent, are strongly recommended.

In this case, etanercept was initially effective in controlling the disease, used in combination with MTX for 1 year, and then as monotherapy for 6 months. Unfortunately, 6 months after MTX discontinuation new signs of anterior chamber inflammation were seen in both eyes, and an impressive flare of polyarticular arthritis was observed soon after. These worsening clinical manifestations prompted the switch from etanercept to another anti-TNF*α*, adalimumab, according to recent compelling evidence [10, 11]. Our experience has been positive, with adalimumab leading to a complete and long-lasting remission of disease. Adalimumab is a fully human subcutaneously administered IgG anti-TNF antibody that binds to TNF*α*, preventing it from activating TNF receptors, thus downregulating the inflammatory reactions associated with several autoimmune diseases.

Experience of adalimumab therapy in JIA comes from the controlled study published by Lovell et al., which showed efficacy and safety in JIA [10]. The results of a recent Japanese multicentre study confirmed adalimumab efficacy in reducing disease activity and maintaining response in paediatric patients with polyarticular JIA [12]. Adalimumab is approved by the U.S. Food and Drug Administration (FDA) for the treatment of rheumatoid arthritis (RA), JIA, ankylosing spondylitis (AS), psoriatic arthritis (PsA), psoriasis, and Crohn's disease. The recommended dose is 24 mg/m^2^ (maximum dose 40 mg), given subcutaneously every other week [13, 14]. Adalimumab is the second TNF antagonist to receive FDA approval for treatment of moderate to severe active polyarticular JIA in patients 4 years and older [15, 16]. Adverse events are rare, mainly consisting of injection site reactions and respiratory tract infections.

The current literature about the use of adalimumab in children with JIA and uveitis who have failed previous therapies reports excellent results. In 2011, Kotaniemi and colleagues, expanding a previous reported cohort [17], showed a significant disease improvement with adalimumab in 94 patients with persistent active JIA and/or associated uveitis [18]. In another study, 39 patients with JIA and refractory uveitis were treated with adalimumab, which, again, proved to be well tolerated and helpful in decreasing ocular inflammatory activity [19].

We previously reported that adalimumab is superior to infliximab in maintaining disease remission in children with refractory chronic uveitis [20]. Moreover, a German study showed better control of uveitis with adalimumab compared with etanercept in a retrospective analysis of data from 18 children and young adults [21]. Finally, a French case series showed that development of new-onset uveitis in patients receiving anti-TNF*α* therapy for rheumatic diseases was higher with etanercept compared with adalimumab or infliximab [22]; in our case, the possibility that etanercept could have triggered anterior chamber ocular activity cannot be excluded.

In conclusion, our experience with adalimumab has been positive, leading to the control of both arthritis and uveitis in a child with psoriatic JIA complicated by uveitis, which was resistant to previous treatments with NSAIDs, DMARDs, and initial TNF inhibitor therapy etanercept.

## Key Points

- JIA is the most common chronic rheumatic disease of childhood, and psoriatic JIA represents up to 10% of all JIA subtypes, with chronic uveitis occurring in 10–15% of children with psoriatic JIA.
- In this case of psoriatic JIA with uveitis, adalimumab treatment brought about complete and sustained remission in both arthritis and uveitis in a child failing previous treatments with NSAIDs, MTX, and anti-TNF*α* therapy.
